# License plate recognition system for complex scenarios based on improved YOLOv5s and LPRNet

**DOI:** 10.1038/s41598-025-18311-4

**Published:** 2025-10-06

**Authors:** Xuanhong Wang, Mingchen Wang, Hongyu Guo, Jiazhen Li, Xian Wang, Yijun Zhang

**Affiliations:** 1 School of Communication and Information Engineering, Xi’an University of Posts and Telecomunications, Xi’an, 710121 China; 2 Test Center, National University of Defense Technology, Xi’an, 710106 China

**Keywords:** YOLOv5s, Object detection, LPRNet, License plate recognition, Triplet attention, Engineering, Mathematics and computing

## Abstract

Traditional license plate recognition (LPR) algorithms perform well in controlled environments but often suffer from accuracy degradation in complex scenarios (such as adverse weather, plate tilt, and varying capture distances) as well as deployment difficulties under hardware constraints. This study proposes a lightweight, end-to-end method for license plate detection and recognition that integrates an improved YOLOv5s with LPRNet. First, we incorporate a Triplet Attention mechanism into the YOLOv5s backbone to enhance feature extraction, more precisely focus on license plate regions and suppress background interference from adverse weather. In the detection post-processing stage, we introduce a Soft-NMS strategy that applies Gaussian-weighted smoothing suppression to overlapping candidate boxes, thereby alleviating the over-suppression of overlapping license plates by traditional NMS and enhancing detection robustness. To address the issue of decreased recognition accuracy caused by license plate tilting, we introduce a Spatial Transformer Network (STN) before the recognition stage to geometrically correct tilted or distorted license plate images, thereby improving recognition accuracy. Experiments conducted on the CCPD2019 and CRPD datasets demonstrate that the proposed method achieves a detection precision of 98.9% and a recognition accuracy of 91.5% on CCPD2019, representing improvements of 3.7% and 8.38% over the baseline YOLOv5s + LPRNet, respectively. The model contains only 7.5 M parameters and 18.1 GFLOPs; it achieves 147 FPS for detection and 0.1138 ms inference time for recognition, indicating potential feasibility for deployment on resource-constrained platforms such as mobile devices and embedded systems.

## Introduction

With the advancement of smart city construction, intelligent transportation systems have developed rapidly. As a key component of intelligent transportation, license plate recognition (LPR) systems play a vital role in ensuring traffic safety, improving traffic efficiency, and enhancing the enforcement and supervision of traffic regulations^1^. Although traditional LPR algorithms have been effectively applied in daily life, their recognition accuracy and level of model lightweighting still need to be further improved when dealing with complex scenarios such as adverse weather, occlusion, and plate tilt. License plate recognition systems designed for complex scenarios have broad application prospects. For example, in sudden traffic accidents or illegal vehicle escape incidents under adverse weather conditions such as rain, snow, or smog, the system is required to quickly and accurately identify the license plate information of the involved vehicle under conditions of image blur, low contrast, and background interference. However, traditional LPR systems perform poorly in such environments. It is therefore urgent to design complex scene LPR algorithms with environmental adaptability, in order to improve the recognition accuracy and stability of the system under non-ideal conditions. In drone inspection scenarios, license plate images are often captured from top-down or oblique viewpoints, which easily leads to significant tilt and geometric distortion. At the same time, the computing resources carried by drones are relatively limited in terms of power consumption, memory, and processing capacity. Traditional models usually have large numbers of parameters and high computational complexity, making them difficult to deploy reliably on such platforms. This can easily cause excessive latency, rapid battery depletion, or video frame loss. These limitations highlight the necessity of model lightweighting. While ensuring recognition accuracy, it is very important to achieve real-time inference in resource-constrained aerial monitoring environments. In real-world monitoring environments such as highway intersections and curved road sections, due to fixed camera installation angles and uncertain vehicle trajectories, vehicles often pass through the monitoring area with a non-frontal posture. Traditional license plate recognition algorithms often fail to handle such tilted or distorted inputs, leading to reduced recognition accuracy and unstable performance. Complex scene LPR algorithms, by introducing plate correction modules and robustness enhancement mechanisms, can effectively handle problems such as tilt, blur, and occlusion, and thus demonstrate stronger applicability and practical value in real-world traffic monitoring applications.

License plate recognition algorithms can be categorized into traditional license plate recognition and deep learning-based license plate recognition. Traditional methods first locate the license plate using features such as text, color, and characters, and then perform character recognition through techniques such as template matching and Support Vector Machines (SVM)^2^. Template matching measures similarity by calculating the correlation coefficient between the input image and the template, and iteratively updates the position and size of the template to minimize the error between the input image and the template. SVM distinguishes different characters by training on large volumes of sample data. A complete deep learning-based license plate recognition task is generally divided into two steps: detecting the license plate and recognizing the characters from the detected region. License plate detection methods can be classified into two types. The first is the two-stage detection method represented by Faster R-CNN^3^which first determines candidate regions and then detects the license plates. This method achieves high detection precision but has a relatively complex model and lower detection speed. The second type includes single-stage detection methods represented by SSD^4^ and the YOLO series^5^which directly predict the location of license plates with higher efficiency. For character recognition, methods such as Convolutional Neural Networks (CNNs)^6^ are used, which automatically learn features from character images and enable efficient character recognition. These algorithms have achieved good performance in standard license plate recognition tasks. However, in complex scenarios or under resource-constrained conditions, the acquired license plate images are often unclear or severely affected by interference, and there is still room for improvement in recognition accuracy and processing speed.

In recent years, significant progress has been made in license plate detection and recognition under complex scenarios. However, most of the existing algorithms focus on improving recognition accuracy to a certain extent, while the practical requirement of balancing both recognition accuracy and real-time performance remains unresolved. Additionally, in real-world traffic environments, the performance of license plate detection and recognition is often affected by adverse weather and plate tilt. For example, Plavac^7^ et al. reported that ALPR recognition accuracy drops significantly under extreme snow or frost interference; field tests by Plate Recognizer showed that when the plate tilt angle exceeds 70°, recognition accuracy decreases markedly, and at 75° the detection precision drops and recognition becomes almost impossible. Therefore, we propose improvements to enhance detection and recognition accuracy under extreme weather conditions and plate tilt. In this paper, the improved YOLOv5s^8^ is adopted as the main network for license plate detection, which achieves a better balance between accuracy and speed. LPRNet^9^ is cascaded for license plate character recognition. Its simplified structure is easy to deploy on resource-constrained devices. The main contributions of this paper are as follows:

- We introduce a Triplet Attention mechanism^10^ at two locations in the backbone of YOLOv5s. This structure enables the network to more precisely focus on license plate regions and key texture information across multi-scale feature maps, thereby improving the feature extraction ability and enhancing license plate detection performance.
- We replace the standard Non-Maximum Suppression (NMS) with a Gaussian-weighted Soft-NMS^11^. Instead of directly discarding low-score bounding boxes when overlapping occurs, this method applies smooth score decay, which mitigates the issue of mis-suppression for overlapping targets and reduces false positives and missed detections of license plates.
- We incorporate a Spatial Transformer Network (STN) before the LPRNet-based recognition module to correct tilted and distorted license plate images prior to recognition, thereby improving license plate recognition accuracy.

Comparative and ablation experiments are conducted on two public datasets, CCPD^12^ and CRPD^13^demonstrating that the improved model proposed in this paper achieves good overall recognition performance under complex environments, with strong stability and robustness.

## Related work

License plate recognition (LPR) tasks based on deep learning are generally divided into two steps. First, the license plate is detected and localized, and then character recognition is performed on the localized plate^14^. To address the challenges of complex lighting conditions and adverse weather, Raghunandan et al.^15^ proposed an image enhancement model based on differential operators. It can effectively enhance the edges and texture details of license plates and improve detection and recognition performance under complex backgrounds and blurry conditions. However, this method still has the problem of false edge enhancement in low-contrast or heavily occluded scenes, and it depends on parameter tuning, with limited generalization ability. Gong et al.^16^ introduced a large-scale dataset containing naturally blurred license plates and developed a dedicated deblurring network. The method significantly improves recognition performance under motion blur and defocus conditions. But the computational complexity of the model may limit its deployment on resource-constrained devices. Tian et al.^17^ proposed KDNet through knowledge distillation and a non-local similarity mechanism. It maintains high detection precision while reducing computational cost and model parameters. However, the introduction of knowledge distillation and the non-local similarity mechanism increases training complexity, requiring more training time and computing resources. This model may not be suitable for running on resource-constrained devices. In addition, Shi et al.^18^ proposed a license plate recognition system based on an improved YOLOv5 and GRU^19^ + CTC^20^, which performs well in both accuracy and efficiency. Nonetheless, the increased model complexity and reliance on large amounts of data remain its limitations. Chen et al. proposed EAND-LPRM^21^ based on SE attention^22^and Wang et al. proposed an improved YOLOv5 scheme^23^ based on CBAM^24^. These methods improve detection and recognition performance by enhancing attention mechanisms. Nevertheless, they generally suffer from complex model structures, high computational cost, and high deployment cost. Zhou et al.^25^ proposed At_LPRNet, which uses a dual-attention mechanism to fuse spatial and channel features and performs feature enhancement for low-quality images. It significantly improves the recognition accuracy of low-quality license plate images (such as blurred, low-resolution, and noise-interfered). However, the dual-attention mechanism and end-to-end network structure may have the problem of increased computational overhead, and whether it can be deployed on embedded devices still needs further verification. Taken together, attention-based methods improve feature extraction but incur high computational cost.

Li et al.^26^ proposed an ultra-lightweight license plate recognition system optimized for embedded microcontrollers, which maintains high recognition accuracy under limited computational resources. Liu et al.^27^ developed Edge–LPR, which achieves efficient operation on edge computing devices through model compression and channel pruning, and attains nearly 97% accuracy on public datasets. Although both models have been optimized for lightweight and speed, their robustness and generalization ability remain insufficient in complex scenarios involving tilted or deformed license plates and background interference. Lightweight deployment-oriented methods are efficient but lack robustness in complex conditions.

Xu et al.^28,29^ proposed EILPR, an end-to-end irregular license plate recognition method that incorporates an automatic perspective alignment module to rectify distorted or tilted license plates before recognition. However, it does not involve modeling optimization in the license plate detection stage. As a result, detection bias may occur in complex backgrounds, affecting the overall end-to-end performance and generalization ability of the system. He et al.^30^ proposed ALP-Net, which is based on a fully convolutional structure and an attention mechanism, avoids character segmentation, improves license plate recognition speed and shows strong generalization ability on multiple datasets. However, this method does not specifically address the problem of geometric distortion caused by license plate tilt and extreme weather. Saputra et al.^14^ proposed a low-light license plate enhancement method based on URetinex-Net, which is applied as a preprocessing step prior to recognition and combined with the TRBA method for license plate recognition. These methods effectively improve plate clarity and recognition accuracy under low-light conditions. However, it primarily targets low-light recognition issues and unsolved challenges such as plate tilt and occlusion caused by rain or snow, and the additional preprocessing step may affect real-time performance.

**Fig. 1:**
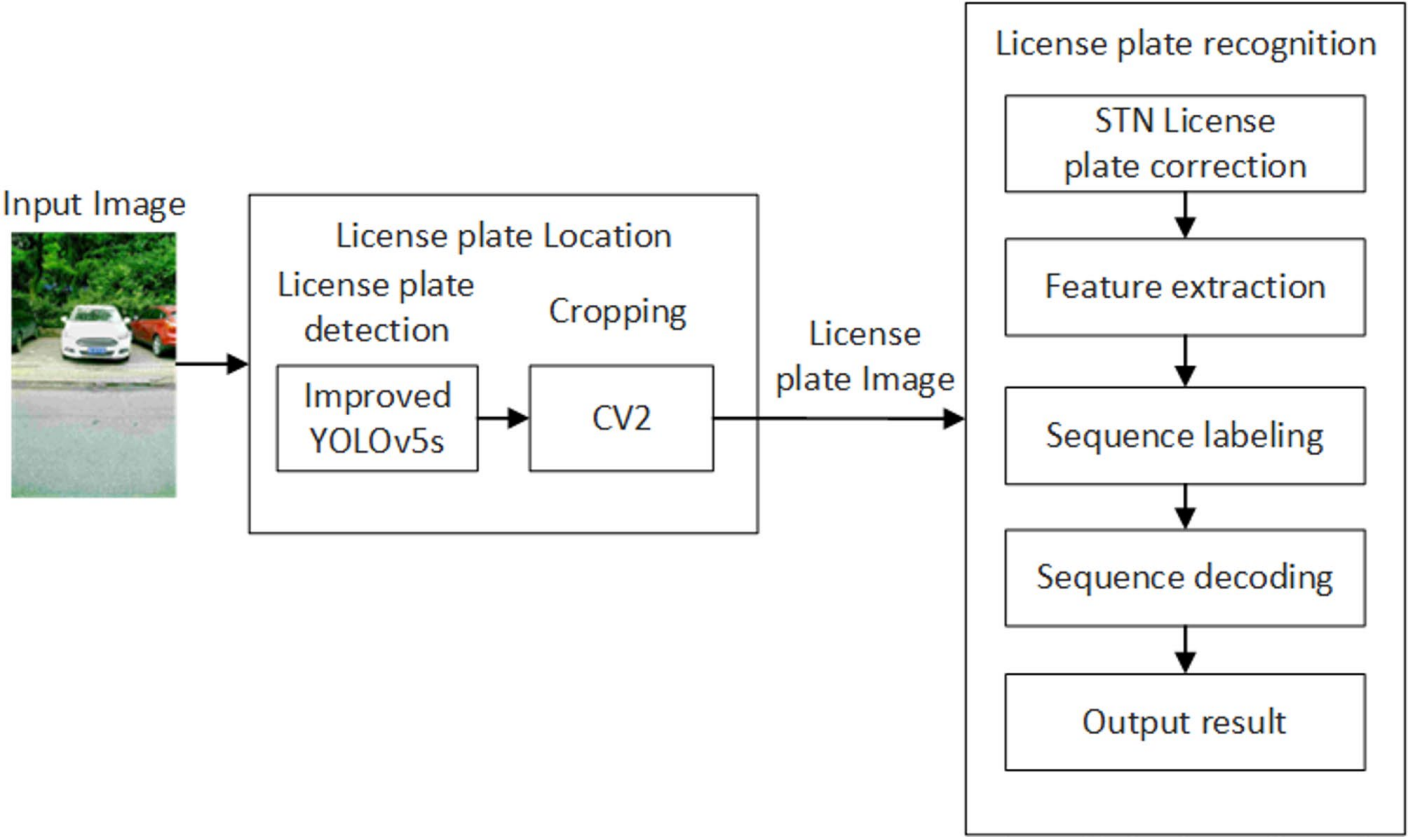
Flowchart of license plate recognition system.

In contrast, our method combines Triplet Attention to enhance multi-scale features with lightweight overhead, Soft-NMS to alleviate mis-suppression in overlapping or occluded plates, and STN to correct tilted license plates before recognition. This integrated design effectively addresses the limitations of existing approaches, achieving a better balance between accuracy, robustness and deployment feasibility. The overall network framework of the license plate recognition system in this paper is shown in Fig. 1. It consists of two parts: license plate localization and license plate recognition. These two parts are combined into an integrated network model through data flow. First, in the license plate localization module, we use an improved YOLOv5s model to detect license plates in the input images, and use OpenCV to draw bounding boxes and crop the license plates. Then, in the recognition network, we use an STN to correct rotated and tilted license plate images, and then use the LPRNet model to complete character recognition. Finally, the recognition result is output.

## Method

### License plate feature extraction enhancement based on triplet attention

YOLOv5 has been widely used in various object detection tasks due to its excellent detection precision and inference speed. The YOLOv5 series includes four model scales: s, m, l, and x. Among them, YOLOv5s has the smallest network depth and channel width. It features the lowest number of parameters and the highest inference efficiency, making it suitable for scenarios with limited computational resources.

The overall structure of YOLOv5s consists of four parts: the input layer, backbone, neck and head. The input layer adopts the Mosaic data augmentation method, which improves sample diversity and enhances the model’s robustness to small objects by cropping, scaling, and randomly stitching multiple images. It also reduces GPU memory consumption. Anchor boxes are generated through adaptive clustering, which further improves the efficiency of candidate box matching. The backbone network is composed of multiple convolutional modules and C3 structures, and uses SPPF (Spatial Pyramid Pooling-Fast) to achieve multi-scale feature extraction. The neck structure integrates FPN and PAN, which are responsible for top-down semantic information transmission and bottom-up localization feature fusion, respectively, enabling cross-scale feature aggregation. Finally, the head performs multi-scale detection on the fused features and outputs the object categories and location information.

The total loss of YOLOv5s consists of three components: bounding box regression loss, objectness loss, and classification loss:

1 $$\:\begin{array}{c}{L}_{total}={\lambda\:}_{box}{L}_{box}+{\lambda\:}_{obj}{L}_{obj}+{\lambda\:}_{cls}{L}_{cls}\end{array}$$

Where $\:{\lambda\:}_{box}$, $\:{\lambda\:}_{obj}$, $\:{\lambda\:}_{cls}$are weighting coefficients.

The bounding box regression loss adopts the Complete IoU (CIoU) loss, defined as:

2 $$\:\begin{array}{c}{L}_{box}=1-IoU+\frac{{\rho\:}^{2}\left(b,{b}^{gt}\right)}{{d}^{2}}+\alpha\:v\end{array}$$

Where $\:IoU$ *i*s the intersection-over-union, $\:{\rho\:}^{2}\left(\text{b},{\text{b}}^{gt}\right)$ is the Euclidean distance between the predicted and ground-truth box centers, d is the diagonal length of the smallest enclosing box covering both predicted and ground-truth boxes, $\:v$ measures aspect ratio consistency, and α is a trade-off parameter.

The objectness loss is computed using binary cross-entropy (BCE):

3 $$\:\begin{array}{c}{L}_{obj}=-\frac{1}{N}\sum\:_{i=1}^{N}\:\left[\begin{array}{c}{y}_{i}{log}\left({p}_{i}\right)+\left(1-{y}_{i}\right){log}\left(1-{p}_{i}\right)\end{array}\right]\end{array}$$

Here, $\:N$ denotes the number of samples (predicted bounding boxes). The term $\:{y}_{i}\in\:\left\{\text{0,1}\right\}$ represents the ground-truth objectness label of the i-th bounding box, where 1 indicates that the box contains an object and 0 otherwise. The term $\:{p}_{i}\in\:\left[\text{0,1}\right]$ denotes the predicted objectness score of the i-th bounding box.

The classification loss also uses BCE:

4 $$\:\begin{array}{c}{L}_{cls}=-\frac{1}{N}\sum\:_{i=1}^{N}\:\sum\:_{c=1}^{C}\:\left[\begin{array}{c}{y}_{ic}{log}\left({p}_{ic}\right)+\left(1-{y}_{ic}\right){log}\left(1-{p}_{ic}\right)\end{array}\right]\end{array}$$

Here, $\:C$ denotes the number of object classes. The term $\:{y}_{ic}\in\:\left\{\text{0,1}\right\}$ represents the ground-truth class label for class $\:c$ of the i-th bounding box, where 1 indicates that the box belongs to class $\:c$ and 0 otherwise, and $\:{p}_{ic}\in\:\left[\text{0,1}\right]$ denotes the predicted probability that the i-th bounding box belongs to class $\:c$.

Since our task contains only a single class, the classification branch is removed and $\:{\lambda\:}_{cls}$ = 0. Consequently, the total loss degenerates to:

5 $$\:\begin{array}{c}{L}_{total}={\lambda\:}_{box}{L}_{box}+{\lambda\:}_{obj}{L}_{obj}\end{array}$$

**Fig. 2:**
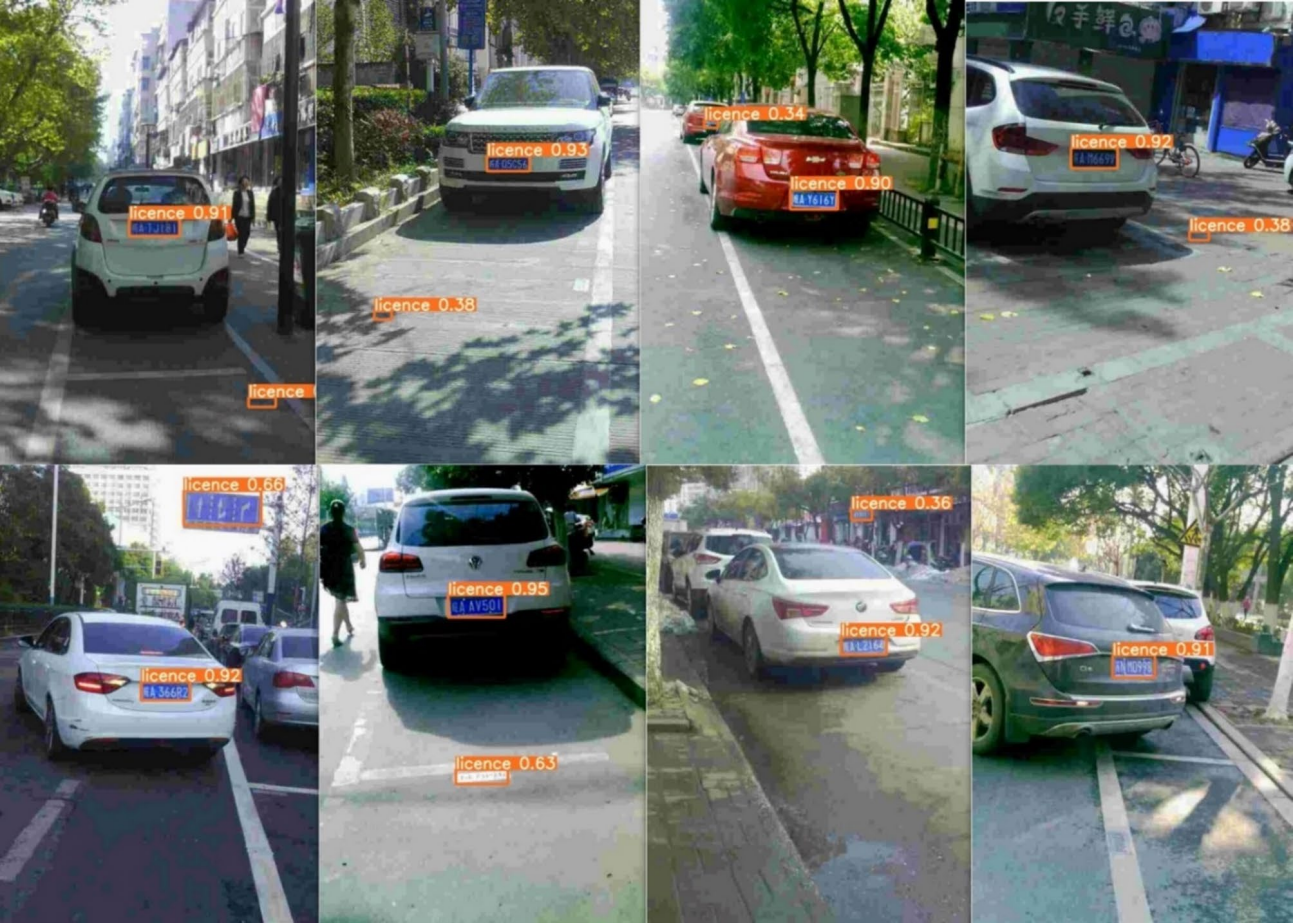
False detection examples of license plate.

In complex traffic scenarios, license plate images are often accompanied by various interfering objects, such as high-brightness vehicle headlights, road markings, manhole covers, and non-license plate elements with rectangular shapes in the background. These distractors share certain visual similarities with real license plates and can easily lead the model to incorrectly detect non-target regions, resulting in false positives and missed detections, as shown in Fig. 2. Therefore, we introduces attention mechanisms into the network to enhance its focus on target regions, reduce false detection rates for license plates, and maintain low computational cost and parameter count.

**Fig. 3:**
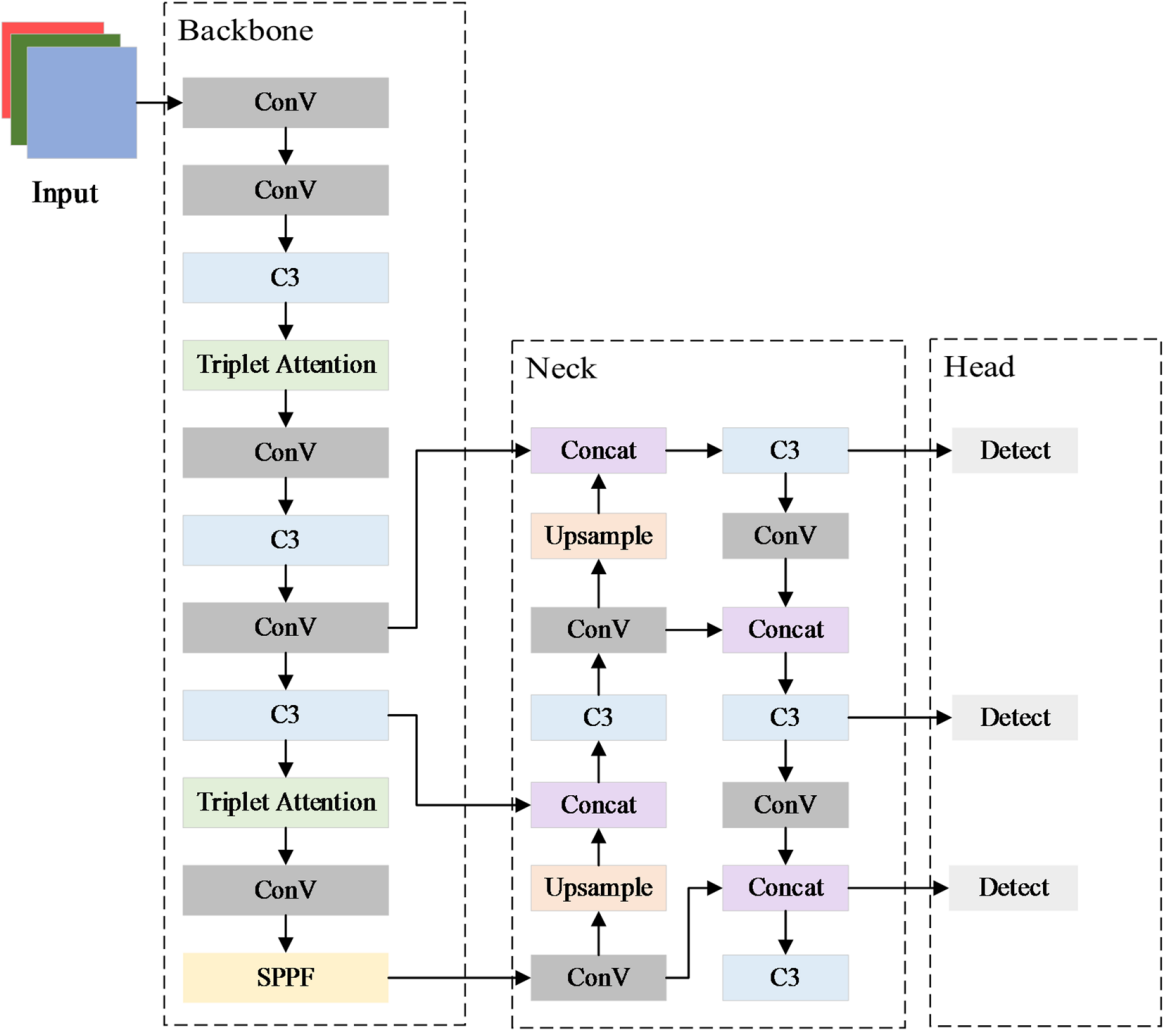
Structure diagram of improved YOLOv5s network. Incorporation of Triplet Attention modules into the 4th and 9th layers of the backbone, enabling joint modeling of spatial and channel dependencies to direct the network’s focus toward critical license plate regions, suppress background interference, and enhance detection performance for small targets.

Triplet Attention is introduced into the backbone network of YOLOv5s to jointly models the dependencies between spatial and channel dimensions. This guides the network to focus on the key regions of license plates, effectively suppresses background interference, and improves the precision and stability of license plate detection. The improved YOLOv5s network structure is shown in Fig. 3. Triplet Attention is introduced at the fourth layer of the backbone network. At this stage, the network has completed preliminary feature extraction, and the feature map size is 1/8 of the original image. The feature map contains rich local information, but may suffer from feature redundancy or imbalance. Triplet Attention is introduced to capture the edge features of small targets and enhance the network’s detection capability for small objects. Subsequently, Triplet Attention is introduced at the ninth layer of the backbone network. At this stage, high-level feature extraction has been completed, and the feature map size is 1/32 of the original image. The feature map contains more abstract semantic information. Triplet Attention is introduced to capture the overall contour and directional characteristics of large targets, and to improve the network’s detection capability for large objects. This design is based on the multi-scale representation capability of convolutional neural networks: shallower layers tend to be more effective for refining local detail features, while deeper layers capture global semantics. Placing Triplet Attention at the 4th and 9th layers leverages these complementary scales, ensuring that both local and global features are effectively enhanced.

**Fig. 4:**
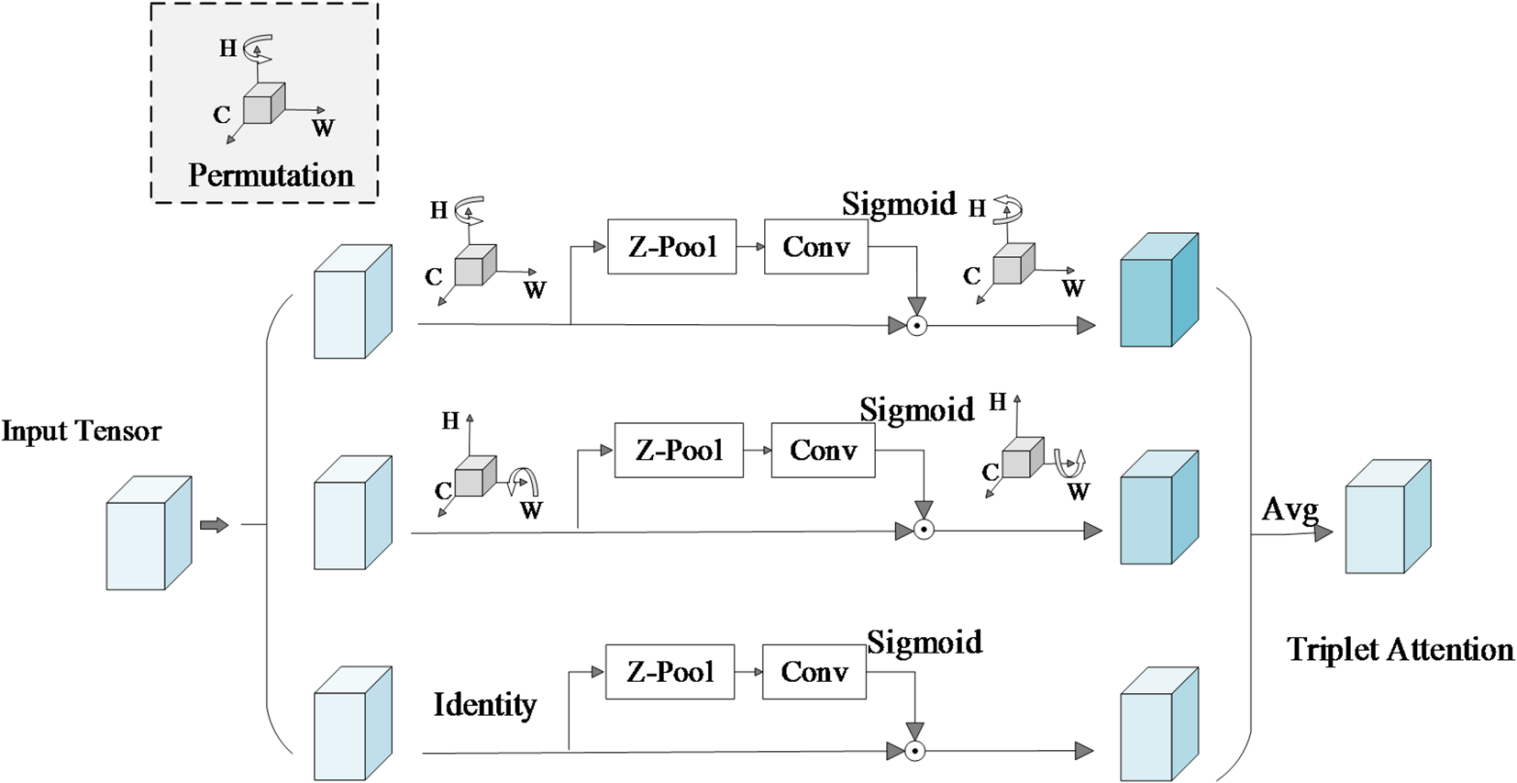
Triplet attention structure diagram. The module cross-dimensional interactions along (H, W), (C, H), and (C, W) to enhance key region responses in license plate detection, while maintaining low computational cost.

Triplet Attention emphasizes the importance of capturing cross-dimensional interactions when computing attention weights, in order to provide rich feature representations. The structure of this module is shown in Fig. 4. The input to this module is a tensor with the shape C×H×W. The entire module consists of three parallel branches, which model the dimensions of (H, W), (C, H), and (C, W), respectively. Each branch performs average pooling and max pooling on the input feature tensor to extract regional saliency information. After concatenation, the features are fed into a convolutional layer with shared parameters for mixed processing. After the convolution, a Sigmoid function is used to generate a normalized attention weight map. This weight map is then applied to the original features through dimension permutation and pointwise convolution (1 × 1 Conv) to complete the weighting operation. Finally, the outputs of the three branches are fused based on residual connections to enhance the network’s response to key regions. This module does not require the introduction of a large number of learnable parameters. It is suitable for complex scenarios in license plate images involving multi-scale targets, occlusion, and tilt. It can effectively enhance the network’s response to license plate regions and improve detection precision.

The computation process of Triplet Attention is as follows:

6 $$\:\begin{array}{c}{M}_{att}=\sigma\:\left({f}_{1\times\:1}\left(Concat\left[AvgPool\left(X\right),MaxPool\left(X\right)\right]\right)\right)\end{array}$$

$\:X$ denotes the input feature map, which contains *C* channels and spatial size is *H × W*. AvgPool(⋅) and MaxPool(⋅) represent average pooling and max pooling operations performed along the specified dimension. Concat[⋅] denotes channel-wise concatenation of the pooling results to form a more informative intermediate representation. $\:{f}_{1\times\:1}$ represents the convolution operation. σ(⋅) denotes the Sigmoid activation function. $\:{M}_{att}$ is the output attention weight map, which indicates the importance of each pixel position.

The formula for applying the attention weights $\:{M}_{att}$ to the original feature map is given as:

7 $$\:\begin{array}{c}{X}^{{\prime\:}}=X\odot\:{M}_{att}\end{array}$$

Here, $\:X$ is the input three-dimensional feature map with *C* channels and a spatial dimension of *H × W*. $\:{M}_{att}$ is the attention map generated through pooling, convolution, and activation operations. It has the same dimensions as $\:X$, the value at each position lies in [0,1], indicating the importance weight of that position.

### Improved soft-NMS non-maximum suppression strategy

To alleviate the problem of incorrect suppression of high-confidence targets by traditional NMS in complex scenarios and to further improve license plate detection performance under such conditions, this paper introduces Soft Non-Maximum Suppression (Soft-NMS) in the post-processing stage of license plate detection. This algorithm attenuates the confidence scores of candidate boxes based on their overlap, instead of directly removing overlapping boxes, which improves detection robustness in scenarios involving target overlap or partial occlusion.

Compared with the suppression mechanism of traditional NMS, which directly removes all low-score boxes when the IoU exceeds a threshold, the Gaussian weighting strategy of Soft-NMS attenuates the candidate box scores using the following formula(4):

8 $$\:\begin{array}{c}{s}_{i}=\left\{\begin{array}{lll}{s}_{i},&\:IoU\left({b}_{top},{b}_{i}\right)<{N}_{t}&\:\:\\\:0,&\:Iou\left({b}_{top},{b}_{i}\right)\ge\:{N}_{t}&\:\:\end{array}\right.\end{array}$$

9 $$\:\begin{array}{c}{s}_{i}={s}_{i}{e}^{-\frac{Iou{\left({b}_{top},{b}_{i}\right)}^{2}}{\alpha\:}},\forall\:{b}_{i}\notin\:D\end{array}$$

Here, $\:{s}_{i}$ denotes the score of the i-th candidate box, $\:{b}_{top}$ represents the box with the highest score, and $\:{b}_{i}$ refers to each remaining boxes excluding $\:{b}_{top}$. α denotes the Gaussian decay parameter that controls the sensitivity of score attenuation with respect to the $\:Iou$. A smaller α results in faster decay for boxes with high overlap, while a larger α produces slower decay. In this work, we set α = 0.5, a commonly used configuration in Gaussian Soft-NMS. Through validation experiments with different values (0.3, 0.5, 0.7), α = 0.5 was found to provide the best trade-off between suppressing highly overlapping boxes and maintaining detection precision and recall. $\:Iou\left({b}_{top},{b}_{i}\right)$ represents the intersection-over-union between $\:{b}_{top}$ and $\:{b}_{i}$.

This strategy makes the score decay more gradual and effectively reduces missed detections caused by occlusion and viewpoint variation. Experimental results show that the introduction of Soft-NMS brings significant advantages in scenarios such as low illumination and long distances.

### Improvements to license plate recognition algorithms

**Fig. 5:**
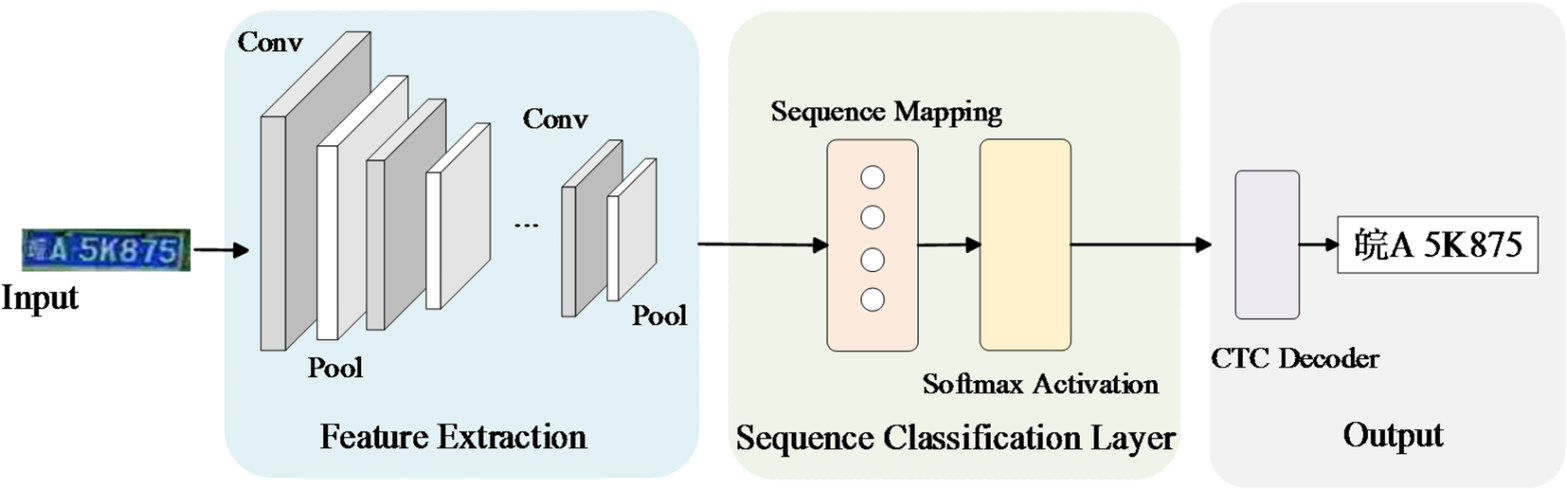
The network structure of LPRNet.

LPRNet is a lightweight and efficient end-to-end license plate recognition network, the network structure is shown in Fig. 5. It has advantages such as a small number of parameters and fast inference speed, making it suitable for embedded deployment scenarios. Unlike the traditional segmentation-recognition pipeline, LPRNet directly takes the entire license plate image as input and extracts high-level semantic features through a convolutional feature extraction module. This design avoids the mis-segmentation problem caused by low resolution or noisy conditions. In terms of architecture, LPRNet uses CNNs to extract spatial features and then converts the two-dimensional feature maps into a character sequence representation through a sequence modeling module. This sequence modeling part adopts a bidirectional long short-term memory network (Bi-LSTM), which captures forward and backward temporal contexts in parallel. It effectively models the dependencies between characters and improves the accuracy of license plate character recognition. Then, a Softmax layer is used to normalize the output features at each time step into a probability distribution over a predefined character set for subsequent decoding. The network uses the Connectionist Temporal Classification (CTC) loss function to train the output character sequence without alignment, enabling weakly supervised end-to-end recognition.

The CTC loss used in LPRNet is defined as:

10 $$\:\begin{array}{*{20}c} {L_{{CTC}} = - logp\left( {y\left| x \right.} \right)} \\ \end{array}$$

where $\:\text{x}$ is the input feature sequence, and $\:\text{y}$ is the target label sequence. The probability $\:p\left( {{\text{y}}\left| {\text{x}} \right.} \right)$ is computed by summing over all possible alignment paths $\:\pi\:$ that correspond to $\:\text{y}$:

11 $$\:p\left( {y\left| x \right.} \right) = \sum\nolimits_{{\pi \: \in \:{\mathcal{B}}^{{ - 1}} \left( y \right)}} {\prod\nolimits_{{t = 1}}^{T} {p_{{\pi \:_{t} }}^{{\left( t \right)}} } }$$

Here, T denotes the length of the input sequence. The sequence $\:\pi\:=({\pi\:}_{1},{\pi\:}_{2},\dots\:,{\pi\:}_{T})$ represents a possible alignment path, which may include blank tokens, that corresponds to the target sequence $\:\text{y}$. The term $\:{p}_{{\pi\:}_{t}}^{\left(t\right)}$ ​ indicates the predicted probability of label $\:{\pi\:}_{t}$ at time step $\:t$. The mapping function $\:\mathcal{B}$ performs a many-to-one transformation by removing repeated labels and blank tokens from the alignment path $\:\pi\:$, thereby producing the final label sequence $\:\text{y}$.

**Fig. 6:**
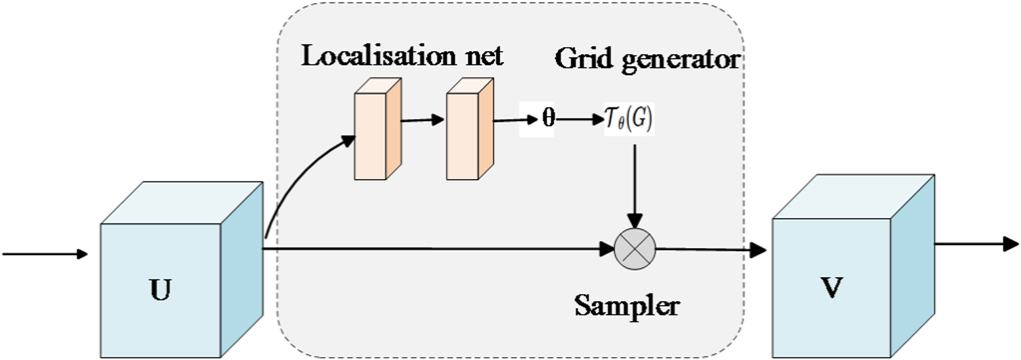
STN transformer networks. The STN adaptively learns affine transformation parameters to correct geometric distortions such as tilt and rotation in license plate images through feature extraction, grid generation, and sampling.

In the license plate recognition network, to enhance the model’s robustness against geometric distortions such as image tilt, rotation, and scale variation, we introduce a Spatial Transformer Network (STN) module before LPRNet, as shown in Fig. 6. STN is a differentiable geometric transformation module that supports end-to-end training and can adaptively perform affine transformation correction on the input image.

Specifically, STN first extracts the spatial features of the input image using a lightweight CNN and then regresses the affine transformation parameters θ. θ corresponds to a 2 × 3 affine transformation matrix, initialized to the identity to avoid excessive geometric distortion in the early stage of training. The parameters are learned from the recognition loss through a differentiable grid sampling process, without the need for explicit supervision. In practice, the learned transformation effectively corrects common in-plane tilts and geometric distortions present in the data, typically within a rotation range of about ± 35°. Next, coordinate transformation function maps the original image coordinates to the target aligned position. Finally, sampler performs bilinear interpolation on the input image based on the transformed coordinates to generate geometrically corrected image features. This mechanism effectively mitigates the negative impact of common non-ideal conditions in license plate images, such as tilt and distortion, on recognition accuracy.

## Experiments and result analysis

### Datasets and experimental environment

The CCPD (Chinese City Parking Dataset) was released by the National Key Laboratory of Intelligent Technology and Systems at Tsinghua University. It aims to provide high-quality training and testing data for license plate detection, recognition, and related computer vision tasks. The dataset was mainly collected from parking lots in Hefei City and contains over 250,000 license plate images, making it one of the largest publicly available license plate datasets. The images cover a variety of complex scenarios, including different lighting conditions, various weather situations (such as rain and snow), license plate tilt, and occlusion. The images in the dataset were captured in real urban parking environments and utilized high-definition surveillance cameras fixed at parking lot entrances, equipped with automatic exposure adjustment and infrared illumination. These characteristics offer high practical relevance for evaluating algorithms in real urban parking environments.

The CRPD dataset was captured from various urban arterial roads and highways in China, encompassing a wide range of traffic environments such as traffic-light intersections, toll stations, and highway ramps, thereby reflecting both high-speed and low-speed driving scenarios. The images were obtained using high-resolution industrial cameras and intelligent traffic cameras from operational traffic monitoring systems, with some devices equipped with anti-glare filters and high-frame-rate modes to capture detailed license plate information from fast-moving vehicles. The dataset includes a diverse set of vehicle types—such as sedans, SUVs, trucks, and buses—and captures complex real-world conditions including strong glare, shadows, dirt, tilt, and multiple plates within a single image. According to the number of license plates present, CRPD is divided into single-, dual-, and multi-plate subsets, providing a valuable benchmark for evaluating a model’s adaptability to varied and challenging environments.

In this study, we selected a total of 6,000 images from the CCPD dataset as the first dataset, with 1,500 images for each of the following conditions: tilted plates, adverse weather, long- or short-distance capture, and standard conditions. The horizontal tilt angles of the plates ranged from 20° to 50°, and the vertical tilt angles ranged from − 10° to 10°. All images had a resolution of 720 × 1160 pixels. The second dataset was selected from the CRPD-single subset, containing 5,000 single-license-plate images with a resolution of 1628 × 1236 pixels. The CRPD-single dataset covers a variety of real-world traffic scenarios, including day/night illumination, different poses, and various weather conditions. Each dataset was divided into training, testing, and validation sets in a 7:2:1 ratio. All experiments were conducted on a Linux operating system. The CPU used was an Intel(R) Core(TM) i9-13900 K (13th Gen), and the GPU was an NVIDIA GeForce RTX 4090 with 24GB of memory. The software environment included CUDA version 11.6 and PyTorch version 1.10.

### Evaluation metrics

In this paper, we adopt objective evaluation metrics to assess the performance of the license plate detection and recognition model, including precision, mean Average Precision (mAP), computational complexity (GFLOPs), and processing speed (FPS). Precision refers to the proportion of correctly predicted positive samples among all samples predicted as positive. It measures the precision of the model’s predictions and is calculated using the following formula:

12 $$\:\begin{array}{c}Precision=\frac{TP}{\left(TP+FP\right)}\end{array}$$

Where TP is the number of true positive samples, and FP is the number of false positive samples.

The mean Average Precision (mAP) combines the performance of both precision and recall, and it provides a comprehensive evaluation of the model’s performance in object detection tasks. Its calculation formula is as follows:

13 $$\:\begin{array}{c}mAP={\int\:}_{0}^{1}\:P\left(R\right)dR\end{array}$$

Where P(R) is the precision as a function of recall R.

GFLOPs refer to the number of floating-point operations (in billions) required by the model, which reflects its computational complexity. FPS (Frames Per Second) is a commonly used speed metric in object detection and image recognition tasks, indicating the number of image frames the model can process per unit time.

## Result analysis

During the training of the improved YOLOv5s license plate detection network, the input image dimensions were uniformly adjusted to 640 × 640 × 3, with a batch size of 16. The model was trained for 300 epochs using the SGD optimizer with an initial learning rate of 0.01, momentum of 0.937, and weight decay of 0.00046875. A cosine decay learning rate schedule was applied, with a final learning rate coefficient of 0.01, and 3 warm-up epochs were used to stabilize gradient updates in the early training stage. To enhance detection robustness, data augmentation techniques including HSV color adjustments (hsv_h = 0.015, hsv_s = 0.7, hsv_v = 0.4), random scaling, translation, and horizontal flipping were employed. These strategies improved the model’s capability to detect license plates under varying illumination, scale, and positional conditions. The purpose of license plate localization is to obtain the region containing the license plate and provide input data for recognition. Therefore, the precision of localization directly affects the effectiveness of recognition.

**Fig. 7:**
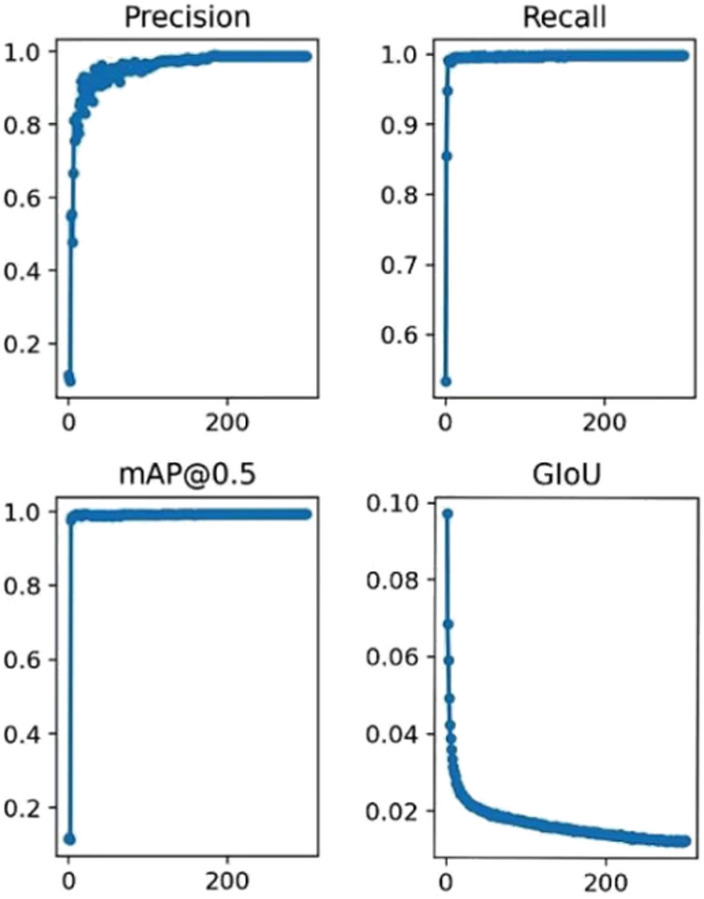
Training results of the improved YOLOv5s model.

After training began, the precision value increased and stabilized at approximately 98.9% by epoch 300. As shown in the bottom-left of Fig. 7, the average precision curve rose quickly during training and finally reached 99.4%. The loss curve on the test set during training is shown in the bottom-right of Fig. 7. Between epochs 200 and 300, the loss gradually stabilized at around 0.01. A model file was generated for the test set at each training epoch. After the overall training was completed, the network was tested using the best.pt file.

**Fig. 8:**
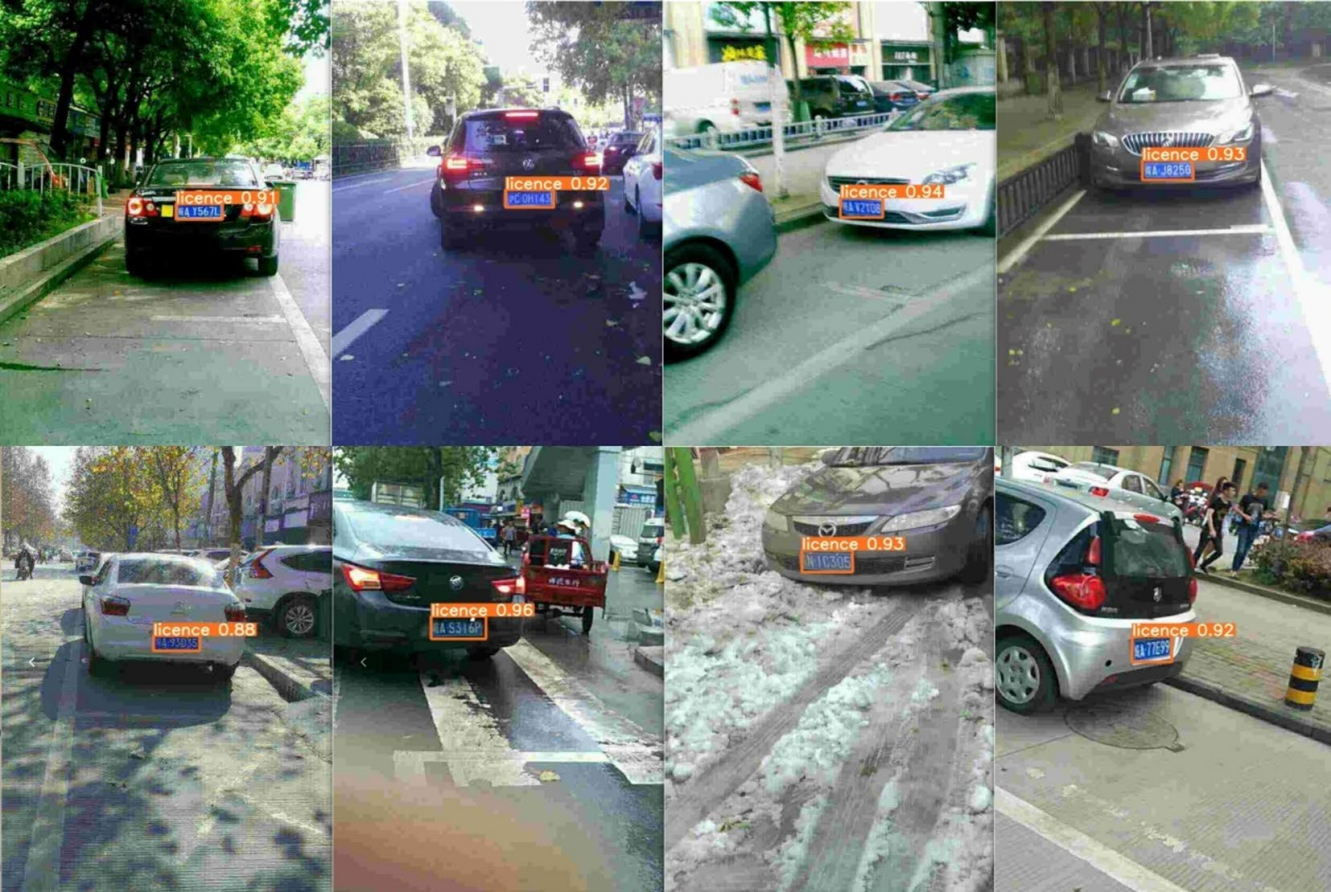
Visualization results of improved YOLOv5s license plate detection.

The test results of the improved detection algorithm are shown in Fig. 8. The license plate regions are detected more accurately both during the day and at night. The model is capable of accurately detecting plates at long distances, as well as under tilt or adverse weather conditions.

Comparative experiments of license plate detection are shown in Tables 1 and 2. On the CCPD dataset, compared with the original YOLOv5s model, the proposed method improves precision from 0.952 to 0.989 in precision and from 0.991 to 0.994 in mAP50, while the parameters increase from 7.26 M to 7.49 M and the model size increases from 13.84 MB to 14.28 MB. Compared with YOLOv7x and KDNet, the proposed model shows slightly higher precision than YOLOv7x and close to KDNet, but with significantly fewer parameters and smaller model size, as well as faster processing speed. Compared with SSD and the two-stage method Faster R-CNN, the proposed model outperforms both in all evaluation metrics. To further verify the generalization ability of the proposed model, comparative experiments were conducted on the CRPD dataset, and the results are shown in Table 2. Although the computational cost and speed are slightly lower than those of YOLOv5s, the proposed model achieves the best results in terms of precision, and mean average precision.

By analyzing the comparative experiments on different datasets, it is evident that the proposed license plate detection model achieves high performance in precision, average precision, computational cost, and speed, while maintaining low complexity. This demonstrates strong performance and efficiency in practical applications. Therefore, the improved model achieves a favorable balance between precision and speed.

**Table 1:** Comparison of license plate detection algorithms on CCPD dataset.

Network	*P*	mAP50	FPS	Params (M)	Model size (MB)	GFLOPs
SSD	0.883	0.860	47	23.90	45.80	24.4
Faster RCNN	0.899	0.875	32	41.10	80.39	43.96
YOLOv7x	0.980	0.993	86	70.78	134.99	188.9
KDNet	0.988	0.991	125	39.59	75.5	107.8
YOLOv5s	0.952	0.991	165	7.26	13.84	17.6
Ours	0.989	0.994	147	7.49	14.28	18.1

**Table 2:** Comparison of license plate detection algorithms on CRPD dataset.

Network	*P*	mAP50	FPS	Params (M)	Model size (MB)	GFLOPs
SSD	0.881	0.853	41	23.90	45.80	23.9
Faster RCNN	0.895	0.869	27	41.10	80.39	43.25
YOLOv7x	0.978	0.993	75	70.78	134.99	188.4
KDNet	0.976	0.994	118	39.59	75.5	107.0
YOLOv5s	0.967	0.992	160	7.26	13.84	16.9
Ours	0.980	0.995	143	7.49	14.28	17.4

Table 3 presents the ablation experiments. The first row represents the baseline model, the second row represents the model with Triplet Attention added to the fourth layer of the backbone, and the third row represents the model with Triplet Attention added to both the fourth and ninth layers. As shown in Table 3, after adding Triplet Attention to two layers, the model is better able to focus on target regions and reduce background interference. The precision reaches 97.8%, and the mAP50 reaches 99.4%, both showing improvements compared to the baseline. Meanwhile, the increase in the number of parameters and model size is relatively small. In addition, when Soft-NMS is used to replace traditional NMS as the post-processing strategy, the precision reaches 96.5% without increasing computational cost. When both enhancements are combined, the model’s precision further improves to 98.9%, and its detection performance becomes more robust in complex scenarios involving occlusion and overlap. The experimental results demonstrate that the proposed detection enhancement strategies are effective and practical for improving detection precision while maintaining model lightweight characteristics.

**Table 3:** Ablation experiments on license plate detection on the CCPD dataset.

Network	*P*	map50	Params (M)	Model size (MB)	GFLOPs
YOLOv5s	0.952	0.991	7.26	13.84	17.6
Triplet attention	0.967	0.993	7.38	14.04	17.9
Two-layer triplet attention	0.978	0.994	7.49	14.28	18.1
Soft-NMS	0.965	0.992	7.26	13.84	17.6
Two-layer triplet attention་Soft-NMS	0.989	0.994	7.49	14.28	18.1

During the training of the LPRNet-based license plate recognition network, the input image dimensions were uniformly adjusted to 94 × 24, with a batch size of 128. The Adam optimizer was employed to train the model for 200 epochs, initial learning rate of 0.0002 and a weight decay of 5 × 10⁻⁵. The learning rate was scheduled to decay progressively over the training process, ensuring stable convergence. During training, the character recognition accuracy steadily increased and reached optimal performance near the final epochs. Upon completion of training, the model parameters corresponding to the best validation accuracy were saved and subsequently used for performance evaluation.

The training and testing results of the improved LPRNet on the CCPD and CRPD datasets are shown in Table 4. Atime refers to the average inference time per image, measured in milliseconds. On both datasets, the recognition accuracy reached 91.45% and 92%, respectively. These results are higher than those of the ALP-Net, CRNN, and EasyPR models, and the proposed model also achieves faster processing speed than the compared models. Compared to the original version of LPRNet, the improved model shows higher accuracy, while the decrease in speed is negligible. In addition, the recognition module is lightweight, containing only 0.91 M parameters with a model size of 1.7 MB. When combined with the improved YOLOv5s detector, the full license plate recognition system (detection + recognition) comprises approximately 8.4 M parameters and a total size of 15.98 MB. The end-to-end processing speed reaches 64 FPS, suggesting that the complete system has the potential to achieve real-time license plate detection and recognition.

**Table 4:** Comparison of license plate recognition performance of different algorithms on CCPD and CRPD datasets.

Network	CCPD		CRPD	
	Accuracy	Atime (ms)	Accuracy	Atime (ms)
EasyPR	0.845	0.126	0.846	0.138
CRNN	0.876	0.142	0.879	0.147
ALP-Net	0.8930	2.2903	0.8931	2.4941
LPRNet	0.8307	0.1128	0.8862	0.1133
Ours	0.9145	0.1138	0.92	0.1108

**Fig. 9:**
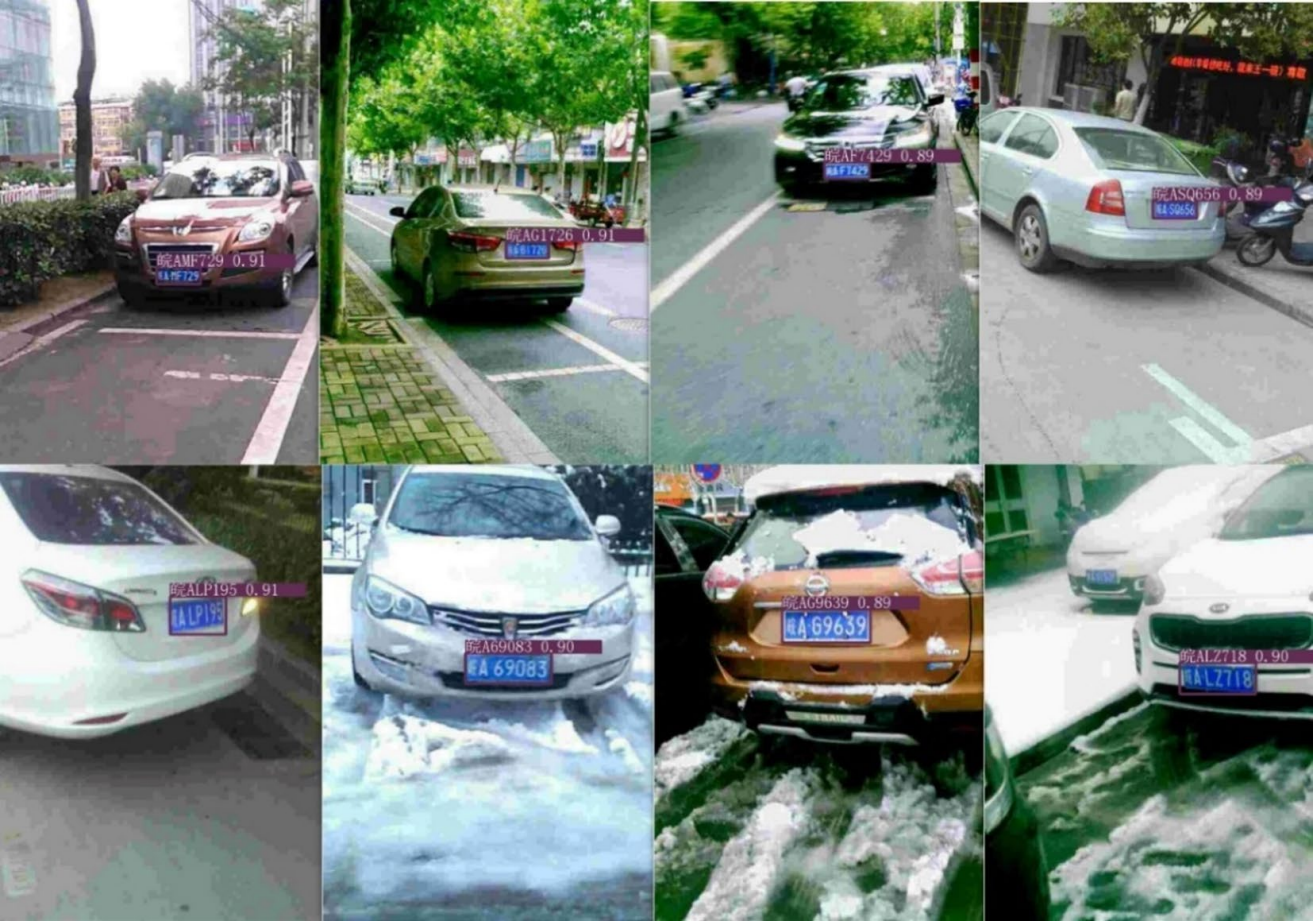
Visualization results of detection and recognition.

Figure 9 shows the detection and recognition results of license plates in complex scenarios using the trained model. The experimental results indicate that the proposed cascaded model demonstrates good recognition performance. The model can accurately recognize license plate images under various conditions, and it meets the requirements of real-time performance, accuracy, lightweight design, and adaptability for complex scenarios.

## Discussion

To further assess the generalization capability of the proposed model, we conducted cross-dataset evaluations between CCPD and CRPD. Specifically, the model trained on CCPD was tested on CRPD, and vice versa. The results are shown in Tables 5 and 6. When trained on CCPD and tested on CRPD, the detection precision decreased from 98.9 to 93.7%, and mAP declined from 99.9 to 98.2%, while recognition accuracy dropped from 91.45 to 89.27%. Conversely, when trained on CRPD and tested on CCPD, detection precision fell from 98.0 to 89.3%, mAP decreased from 99.5 to 94.2%, and recognition accuracy declined from 92.0 to 85.31%.

**Table 5:** License plate detection performance under different train-test combinations.

Training dataset	Test dataset	*P*	mAP50	FPS
CCPD	CCPD	0.989	0.994	147
CCPD	CRPD	0.937	0.982	156
CRPD	CRPD	0.980	0.995	143
CRPD	CCPD	0.893	0.942	138

**Table 6:** License plate recognition performance under different train-test combinations.

Training dataset	Test dataset	Accuracy	Atime (ms)
CCPD	CCPD	0.9145	0.1138
CCPD	CRPD	0.8927	0.1143
CRPD	CRPD	0.9200	0.1108
CRPD	CCPD	0.8531	0.1106

This performance degradation can be primarily attributed to differences in dataset characteristics, including license plate styles, image resolutions, and background complexities. Notably, the performance drop was more pronounced in the CRPD-to-CCPD transfer. This may be because CCPD contains a higher proportion of samples involving long-distance captures, rotated or tilted plates, and adverse weather conditions, leading the model to learn feature patterns that are more adaptive to complex scenarios during training. However, such features are not the primary discriminative cues in CRPD, which predominantly comprises near-distance, high-resolution plates. As a result, a model trained solely on CRPD lacks targeted optimization for the more challenging and irregular images in CCPD, thereby causing a sharper performance decline.

Beyond dataset distribution disparities, the cross-dataset generalization ability of the model still has room for improvement. While high accuracy is achieved on the source dataset, adaptability to unseen domains remains limited. Future work will consider incorporating training data from more diverse sources and exploring domain adaptation techniques to enhance robustness across different real-world environments.

**Fig. 10:**
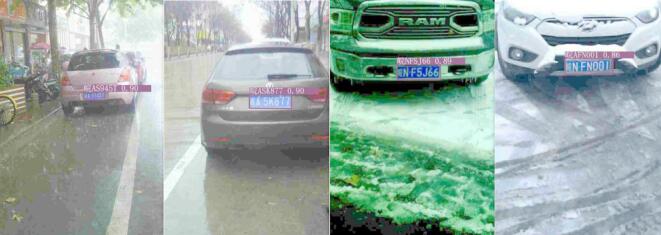
Examples of license plate recognition failures.

In addition, we further analyzed recognition failure cases to better understand the model’s limitations, as shown in Fig. 10. The most common errors involve confusing visually similar characters, such as misrecognizing “D” as “0” or “5” as “S.” These mistakes often arise under conditions of slight blurring or low resolution. Such confusions suggest that the model still faces challenges in fine-grained character discrimination, which may be related to imbalanced distributions of similar characters in the training data as well as limited discriminative power of deep features at small scales.

In future work, we plan to enrich the training set with more diverse samples of easily confusable characters and explore advanced loss functions or contrastive learning strategies to improve character-level robustness. These improvements are expected to further reduce misclassification rates and enhance system stability in practical applications.

## Conclusions

To address the limitations of traditional license plate recognition methods in terms of accuracy and speed under complex scenarios, this paper proposes an end-to-end deep learning model for license plate localization and recognition. We improve the YOLOv5s-based detection algorithm by integrating the Triplet Attention mechanism into the network structure. In the post-processing stage of detection, the Soft-NMS mechanism is introduced, where a Gaussian function is used to attenuate the confidence scores of overlapping candidate boxes. This helps alleviate the mis-suppression problem caused by occlusion or overlap in traditional NMS, and further enhances detection precision. In addition, to reduce the negative impact of tilted or distorted plates on recognition performance, a license plate correction module is added before the recognition network. Finally, an improved cascaded YOLOv5s + LPRNet recognition network is constructed to achieve license plate recognition without the need for character segmentation. Experimental results show that the improved model outperforms traditional approaches. On the CCPD dataset, it achieves a detection precision of 98.9% and a recognition accuracy of 91.45%, while maintaining a real-time processing speed in terms of FPS. In summary, the proposed method significantly improves recognition performance in complex scenarios while maintaining a lightweight architecture, demonstrating strong potential for practical engineering applications. Future work will focus on deploying the model to embedded platforms to build an efficient and stable license plate recognition system for complex edge environments.

## Data Availability

The datasets used in this paper are publicly available and can be accessed at https://github.com/detectRecog/CCPD and https://github.com/yxgong0/CRPD.
